# The role of glycolysis and mitochondrial respiration in the formation and functioning of endothelial tip cells during angiogenesis

**DOI:** 10.1038/s41598-019-48676-2

**Published:** 2019-08-30

**Authors:** Bahar Yetkin-Arik, Ilse M. C. Vogels, Patrycja Nowak-Sliwinska, Andrea Weiss, Riekelt H. Houtkooper, Cornelis J. F. Van Noorden, Ingeborg Klaassen, Reinier O. Schlingemann

**Affiliations:** 1 Ocular Angiogenesis Group, Department of Ophthalmology, Amsterdam Cardiovascular Sciences and Cancer Center Amsterdam, Amsterdam UMC, University of Amsterdam, Meibergdreef 9, Amsterdam, The Netherlands; 2 Department of Medical Biology, Amsterdam Cardiovascular Sciences and Cancer Center Amsterdam, Amsterdam UMC, University of Amsterdam, Meibergdreef 9, Amsterdam, The Netherlands; 3 Molecular Pharmacology Group, School of Pharmaceutical Sciences, Faculty of Sciences, University of Lausanne and University of Geneva, Geneva, Switzerland; 4 Laboratory Genetic Metabolic Diseases, Amsterdam Gastroenterology and Metabolism, Amsterdam Cardiovascular Sciences, Amsterdam UMC, University of Amsterdam, Meibergdreef 9, Amsterdam, The Netherlands; 5 Department of Genetic Toxicology and Cancer Biology, National Institute of Biology, Ljubljana, Slovenia; 6 Department of Ophthalmology, University of Lausanne, Jules-Gonin Eye Hospital, Fondation Asile des Aveugles, Lausanne, Switzerland

**Keywords:** Cell growth, Mechanisms of disease, Angiogenesis

## Abstract

During sprouting angiogenesis, an individual endothelial tip cell grows out from a pre-existing vascular network and guides following and proliferating stalk cells to form a new vessel. Metabolic pathways such as glycolysis and mitochondrial respiration as the major sources of adenosine 5′-triphosphate (ATP) for energy production are differentially activated in these types of endothelial cells (ECs) during angiogenesis. Therefore, we studied energy metabolism during angiogenesis in more detail in tip cell and non-tip cell human umbilical vein ECs. Small interfering RNA was used to inhibit transcription of glycolytic enzymes PFKFB3 or LDHA and mitochondrial enzyme PDHA1 to test whether inhibition of these specific pathways affects tip cell differentiation and sprouting angiogenesis *in vitro* and *in vivo*. We show that glycolysis is essential for tip cell differentiation, whereas both glycolysis and mitochondrial respiration occur during proliferation of non-tip cells and in sprouting angiogenesis *in vitro* and *in vivo*. Finally, we demonstrate that inhibition of mitochondrial respiration causes adaptation of EC metabolism by increasing glycolysis and vice versa. In conclusion, our studies show a complex but flexible role of the different metabolic pathways to produce ATP in the regulation of tip cell and non-tip cell differentiation and functioning during sprouting angiogenesis.

## Introduction

Blood vessel sprouts are characterized by leading tip cells that grow out from a pre-existing vascular network, and by trailing stalk cells. The tip cell at the forefront of the sprouting vessel is attracted by microenvironmental signals and navigates by following gradients of angiogenic factors, such as vascular endothelial growth factor (VEGF), that regulate tip and stalk cell functions^1,2^. Recently, the importance of metabolic regulation in the differential activation of these angiogenic endothelial cells (ECs) was shown^3^.

**Figure 1:**
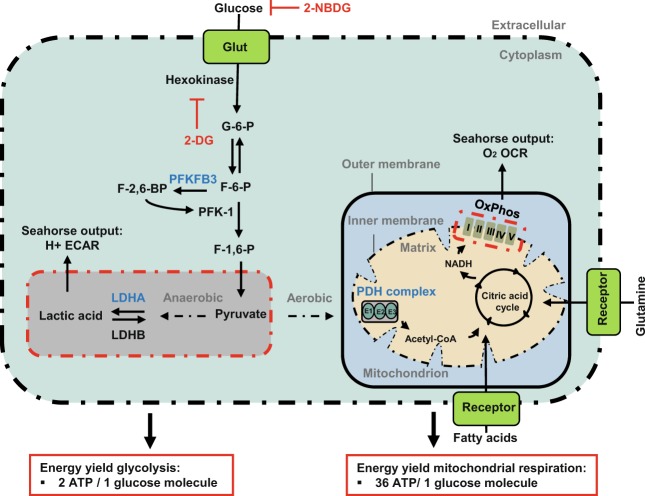
Schematic overview of glycolysis and mitochondrial respiration. Glycolysis and mitochondrial respiration are two major energy-yielding pathways. Glucose is converted into pyruvate in the glycolytic pathway. The fate of pyruvate is dependent on many factors, of which oxygen availability is important. In anaerobic conditions, pyruvate is converted into lactate by **LDHA** in the cytoplasm. LDHB converts lactate into pyruvate. **PFBFB3** enzymes generate fructose-2,6-biphosphate (F2,6P_2_), an allosteric activator of 6-phosphofructo-1-kinase (PFK-1) that is involved in one of the rate-limiting steps of glycolysis by the conversion of fructose-6-phosphate (F6P) to fructose-1,6-biphosphate (F1,6P_2_). ECAR is a measure of lactic acid levels, generated by anaerobic glycolysis. In aerobic conditions, pyruvate enters the citric acid cycle via the **PDH** complex, and is catabolized by oxidative phosphorylation, and ATP is produced by ATP synthase (complex Ѵ). OCR is a measure of oxygen utilization in cells and is an indicator of mitochondrial function. The conversion of glucose into lactate generates 2 ATP per glucose molecule as compared to 36 ATP per glucose molecule when the oxidative phosphorylation is used. 2-NBDG; 2-[N-(7-nitobenz-2-oxa-1,3-diazol-4-yl)-amino]-2-deoxy-D glucose. 2-DG; 2-deoxyglucose. Glut; glucose transporters. G-6-P; glucose-6-phosphate. F-6-P; fructose-6-phosphate. PFKFB3; 6-phosphofructo-2-kinase/fructose-2,6-biphosphatase 3. F-2,6-BP; fructose-2,6-biphosphate. F-1,6-P; fructose-1,6-phosphate. LDHA; lactate dehydrogenase A. LDHB; lactate dehydrogenase B. PDH; pyruvate dehydrogenase. NADH; nicotinamide adenine dinucleotide. FADH_2_; flavin adenine dinucleotide. H^+^; proton. OxPhos; oxidative phosphorylation. ECAR; extracellular acidification rates. OCR; oxygen consumption rates. ADP; adenosine 5′-diphosphate. ATP; adenosine 5′-triphosphate.

In animal and *in vitro* models, it has been demonstrated that differentiated ECs are characterized by glycolytic formation of adenosine 5′-triphosphate (ATP) for energy production and have mitochondrial respiration as the secondary source of ATP. It has been shown that ECs increase glycolysis in response to angiogenic activation, a condition with metabolic characteristics similar to proliferative cancer cells^4–10^. Glycolysis is not efficient for ATP production because only 2 ATP molecules per glucose molecule are generated, whereas mitochondrial respiration produces 36 ATP molecules per glucose molecule (Fig. 1). The need for glycolysis in cancer cells was discovered recently because lactate can be used to produce building blocks for biosynthesis, which is needed in proliferating cells^11,12^. However, upregulation of mitochondrial respiration and thus oxidative phosphorylation can also occur in cancer cells when needed, indicating flexibility of cancer cells in ways to generate ATP^8–10,13^. Moreover, recent studies have suggested that mitochondrial respiration is essential for angiogenic capacity and homeostasis of the endothelium, although ECs are considered to have a glycolytic phenotype^14–16^. Mitochondria in lung ECs have been shown to contribute to reactive oxygen species (ROS)-dependent VEGF production and it has been demonstrated as well that proliferating ECs depend on mitochondrial respiration^17–19^. Collectively, these findings suggest that the metabolism plays an important role in EC differentiation and functioning during angiogenesis. However, how critical glycolysis and mitochondrial respiration are for EC differentiation and EC functions in angiogenesis remains to be elucidated.

In the present study, we apply small interfering RNA (siRNA) against the glycolytic genes 6-phosphofructo-2-kinase/fructose-2,6-biphosphatase 3 (*PFKFB3*), lactate dehydrogenase A (*LDHA*) and the mitochondrial gene pyruvate dehydrogenase E1 alpha 1 subunit (*PDHA1*) to test whether inhibition of glycolysis and/or mitochondrial respiration affects tip cell and/or non-tip cell differentiation and sprouting angiogenesis *in vitro* and *in vivo* (a schematic overview of the relevant enzymes is shown in Fig. 1). This has the following rationale:

PFKFB enzymes generate fructose-2,6-biphosphate (F2,6P_2_), an allosteric activator of 6-phosphofructo-1-kinase (PFK-1) that is involved in one of the rate-limiting steps of glycolysis by the conversion of fructose-6-phosphate (F6P) to fructose-1,6-biphosphate (F1,6P_2_)^20^. A recent study shows that among all isoforms of PFKFB, **PFKFB3** is the most upregulated isoform in stimulated ECs *in vitro* as well as *in vivo*^21^. PFKFB3 is a critical enzyme for glycolysis in ECs and silencing of its expression partially reduces glycolysis and impairs EC migration, proliferation, and induces defects in the formation of filopodia^5,22^.

The **LDHA** enzyme, which catalyzes the conversion of pyruvate into lactate, plays a vital role in glycolysis. Upregulated *LDHA* expression is characteristic for rapidly growing cells, and inhibition of *LDHA* expression impairs vascularization and suppresses tumor cell growth^8,23–26^. LDHA has been shown to be essential for microvascular ECs by enhancing VEGF production in these cells during angiogenesis^27,28^.

The pyruvate dehydrogenase (PDH) complex is the gatekeeper enzyme between glycolysis and mitochondrial respiration and plays an important role in the channeling of pyruvate into aerobic ATP formation by mitochondrial respiration^29^. The E1α subunit of the PDH complex contains the E1 active site that plays a key role in the enzymatic activation of **PDHA1**^30–33^. Various types of cancer cells have been shown to adapt their metabolism by increasing glycolysis after inhibition of PDHA1 activity which reduces the flow of pyruvate into mitochondrial respiration^34^.

The relative contribution of glycolysis and mitochondrial respiration in ATP production in endothelial tip cells and non-tip cells has not been investigated, because thus far discrimination between tip cells and the other angiogenic phenotypes has not been possible *in vitro*. We have developed an *in vitro* approach based on identification and isolation of CD34^+^ tip cells and CD34^−^ non-tip cells in EC cultures^1,35^. The purpose of the present study was to investigate the paradoxical role of mitochondria in ECs and to shed light on the potential regulatory roles of glycolysis and mitochondrial respiration in tip cells and non-tip cells during angiogenesis.

## Results

### Glycolysis is necessary for tip cell differentiation

**Figure 2:**
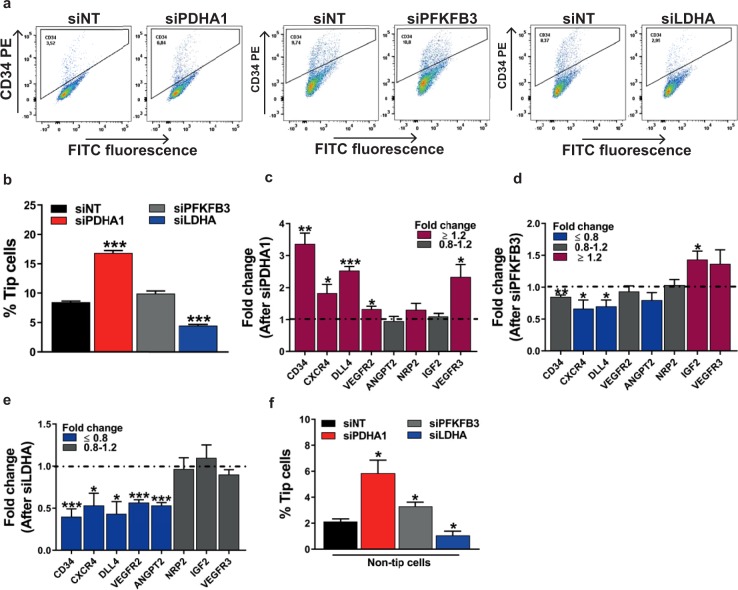
Effects of inhibition of glycolysis or mitochondrial respiration on endothelial tip cell differentiation in HUVEC cultures. (**a**) FACS measurements of tip cells with the use of PE-conjugated anti-CD34 antibody after inhibition of the expression of *PDHA1*, *PFKFB3*, or *LDHA* with siRNA in HUVECs. Inhibition of the expression of the mitochondrial gene *PDHA1* (si*PDHA1* versus siNT) in HUVEC cultures increased relative tip cell numbers (**b**) and increased mRNA expression levels of 5 out of 8 tip cell-specific genes (**c**) at 72 h after siRNA transfection. No effects on relative tip cell numbers were found after inhibition of *PFKFB3* expression (**b**), neither did it cause a uniform directional increase or decrease of tip cell-specific mRNA levels (**d**) at 72 h after siRNA transfection. Silencing of the expression of the glycolytic gene *LDHA* reduced the percentages of tip cells (**b**) and decreased the expression of 5 out of 8 tip cell-specific genes (**e**) at 72 h after siRNA transfection. (**f**) FACS-sorted HUVECs with the use of PE-conjugated anti-CD34 antibody. Inhibition of *PDHA1* or *PFKFB3* expression, respectively, in isolated fractions of non-tip cells increased relative tip cell numbers, whereas silencing of *LDHA* expression diminished the relative tip cell numbers. Percentage of tip cells was determined as described in the methods section. Results are shown as means ± SEM of experiments with HUVECs of at least 3 donors. *P < 0.05, **P < 0.01, and ***P < 0.001 as compared to siNT (Unpaired Student’s t-test).

Metabolic pathways have been identified as major regulators of angiogenesis, and a metabolic switch has been associated with angiogenic EC differentiation^3,5^. To study these metabolic aspects of ECs during angiogenesis further, we inhibited mRNA levels of the genes *PFKFB3*, *LDHA*, or *PDHA1* to determine whether inhibition of glycolysis and/or mitochondrial respiration affects tip cell and/or non-tip cell differentiation (Fig. 2a). Inhibition of expression of *PDHA1*, a mitochondrial respiration enzyme, increased the fraction of tip cells from 5.3% to 10.6% (2-fold; Fig. 2b) and increased the expression levels of 5 out of 8 tip cell-specific genes, including *CD34* (3.4-fold) at 72 h (Fig. 2c). Silencing of expression of the glycolysis gene *PFKFB3* did not have an effect on the percentage of tip cells (Fig. 2b), nor did it result in a consistent directional change of tip cell-specific mRNA levels. mRNA levels of 2 out of 8 tip cell-specific genes (*IGF2*^36^ and *VEGFR3*) were increased, whereas mRNA levels of 3 out of 8 tip cell-specific genes (*CD34 (1*.*1-fold)*, *CXCR4*, and *DLL4*) were decreased (Fig. 2d). Inhibition of expression of the glycolytic gene *LDHA* reduced the percentage of tip cells from 9% to 4.7% (1.9-fold; Fig. 2b), and decreased the expression levels of 5 out of 8 tip cell-specific genes in the mixed EC population, including *CD34* (by 1.6-fold) (Fig. 2e). Western blotting analysis of CD34 protein after transfection of siRNA against *PDHA1*, *PFKFB3*, and *LDHA*, respectively, confirmed these findings at the protein level (Supplementary Fig. S1a).

Non-tip cells are able to differentiate into tip cells^19,37^. To investigate whether a metabolic switch is involved in this transformation, we inhibited the expression of *PDHA1*, *PFKFB3*, or *LDHA* with siRNA in an isolated fraction of non-tip cells, and measured the formation of new tip cells at 72 h after transfection. Inhibition of *PDHA1* or *PFKFB3* expression in non-tip cells increased the fraction of newly formed tip cells by 2.9-fold and 1.6-fold, respectively, as compared to non-tip cells that were transfected with siNT. In contrast, inhibition of *LDHA* expression decreased the percentage of tip cells by 2.1-fold as compared to non-tip cells treated with siNT (Fig. 2f).

These data show that inhibition of *PDHA1* stimulates the formation of tip cells in the total EC population and induces a switch to the tip cell phenotype in non-tip cells. Inhibition of *PFKFB3* had no effect on the number of tip cells in the total EC population, whereas inhibition of *PFKFB3* mRNA expression in a culture of isolated non-tip cells induced the tip cell phenotype. Finally, inhibition of *LDHA* in both the total EC population and the isolated non-tip cell population reduced the percentage of tip cells, indicating that *LDHA*, which produces lactate from pyruvate, is necessary for tip cells to maintain their phenotype and is involved in the transformation of non-tip cells into tip cells.

### Glycolysis and mitochondrial respiration are essential for HUVEC proliferation, whereas tip cells need glucose consumption for mitochondrial ATP production for cell survival

As metabolism affects proliferation and survival of differentiated ECs^38,39^, we investigated whether inhibition of the expression of *PDHA1*, *PFKFB3*, or *LDHA* has effects on tip cell/non-tip cell survival and/or proliferation of non-tip cells.

**Figure 3:**
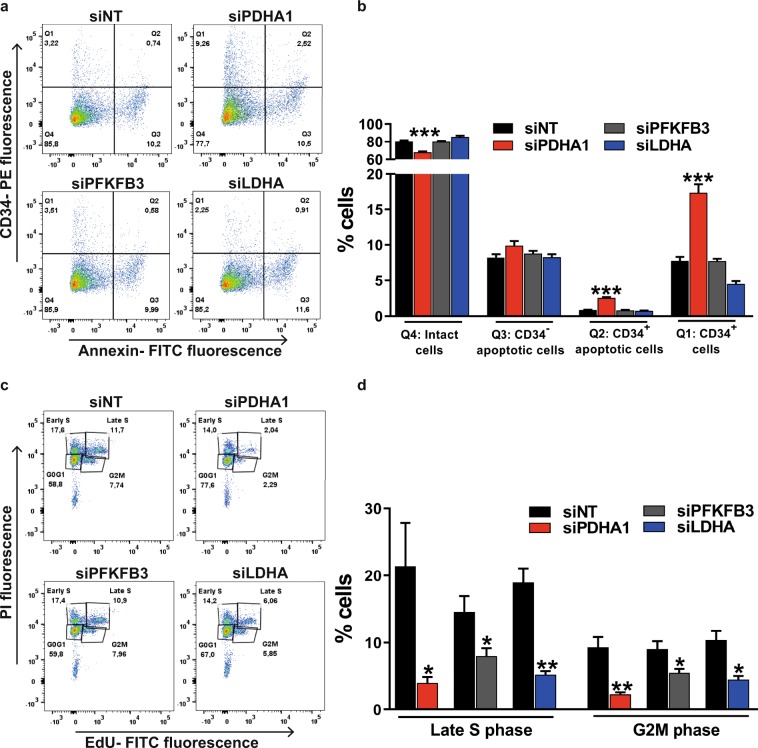
Effects of the inhibition of glycolysis or mitochondrial respiration on survival of endothelial tip cells and non-tip cells and on proliferation of ECs in HUVEC cultures. (**a**) Apoptotic cell death of tip cells and non-tip cells after flow cytometric analysis of HUVECs stained with both PE-conjugated anti-CD34 antibody and FITC-conjugated annexin V antibody at 72 h after siRNA transfections against *PDHA1*, *PFKFB3*, or *LDHA*, respectively. Four quadrants (Q) representing: viable tip cells (Q1), apoptotic tip cells (Q2), apoptotic non-tip cells (Q3), and viable non-tip cells (Q4). (**b**) Inhibition of *PDHA1* expression increased the percentage of apoptotic tip cells, whereas no effect was found in non-tip cells. Inhibition of *PFKFB3* or *LDHA* expression, respectively, did not alter percentages of apoptotic tip cells and non-tip cells. (**c**) Cell proliferation assay of HUVECs expressed as percentages of non-proliferating cells in the G0/G1 phase, and proliferating cells in early S phase, late S phase and G2M phase after flow cytometric analysis of fluorescence of incorporated EdU at 72 h after siRNA transfections against *PDHA1*, *PFKFB3*, or *LDHA*, respectively. (**d**) Inhibition of the expression of *PDHA1*, *PFKFB3*, or *LDHA* lowered percentages of proliferating HUVECs in the late S phase and G2M phase. Results are shown as means ± SEM of experiments with HUVECs of at least 3 donors. *P < 0.05, **P < 0.01, and ***P < 0.001 as compared to siNT (Unpaired Student’s t-test).

First, we tested whether inhibition of *PDHA1*, *PFKFB3*, or *LDHA* expression affects the survival of tip cells or non-tip cells. For this purpose, apoptosis in isolated fractions of tip cells and non-tip cells was measured at 72 h after siRNA transfection (Fig. 3a). Inhibition of *PDHA1* expression increased the percentage of apoptotic tip cells from 0.9% to 2.6%, and showed no effect on non-tip cells. No effects on apoptosis were found in sorted cultures of HUVECs transfected with siRNA against *PFKFB3* or *LDHA*, as compared to controls (Fig. 3b). To verify the importance of glucose oxidation for tip cell survival, we blocked the channeling of pyruvate into mitochondrial respiration with the use of 2-cyano-3-(1-phenyl-1H-indol-3-yl)-2-propenoic acid (UK5099) and measured the effect on the percentage of tip cells. Treatment of HUVECs with UK5099 reduced the number of tip cells at 24 h after treatment by 30% (Supplementary Fig. S1b).

Next, we determined whether glycolysis and/or mitochondrial respiration are necessary for the proliferation of HUVECs (Fig. 3c). Percentages of non-proliferating HUVECs in the G0/G1 phase were increased after inhibition of expression of *PDHA1* (from 57% to 73%) or *PFKFB3* (from 60% to 65%) (Supplementary Fig. S1c,d, respectively). No effects on non-proliferating HUVECs in the G0/G1 phase were found after inhibition of expression of *LDHA* (Supplementary Fig. S1e). Percentages of proliferating HUVECs in the late S phase (EdU-positive cells) and the G2M phase were reduced after inhibition of expression of *PDHA1* (from 21% to 4% and from 9% to 2%, respectively), *PFKFB3* (from 15% to 8% and from 9% to 6%, respectively), or *LDHA* (from 19% to 5% and from 10% to 5%, respectively) as compared to controls (Fig. 3d).

These findings indicate that tip cells need glucose consumption for mitochondrial ATP production for cell survival, and that glycolysis as well as mitochondrial respiration are essential for HUVEC cell proliferation.

### HUVECs adapt their metabolism to microenvironmental circumstances

In response to angiogenic activation, differentiated ECs are known to exhibit metabolic flexibility, characterized by the ability to respond or adapt to conditional changes in metabolic demand by shifting between substrates and/or metabolic pathways^5,19^. To further investigate the metabolic flexibility of ECs, we studied glycolysis and mitochondrial respiration after siRNA inhibition of *PDHA1*, *PFKFB3*, or *LDHA* in more detail in HUVECs.

Tip cells have lower *PDHA1* and *LDHA* expression levels as compared to non-tip cells, whereas *PFKFB3* mRNA expression levels were similar in tip cells and non-tip cells (Supplementary Fig. S1f)^35^. To test the metabolic flexibility of ECs, we determined whether inhibition of the expression of each of these genes in mixed EC populations affects expression levels of the other two genes and/or total ATP levels at 72 h after transfection. Inhibition of *PDHA1* expression increased the expression levels of the glycolytic gene *PFKFB3* (1.4-fold) and did not have an effect on *LDHA* expression levels. After *PFKFB3* silencing, no differences in *LDHA* and *PDHA1* mRNA expression levels were found. Inhibition of *LDHA* expression downregulated expression levels of *PFKFB3* (1.5-fold) and showed no effect on *PDHA1* mRNA expression levels (Supplementary Fig. S1g).

**Figure 4:**
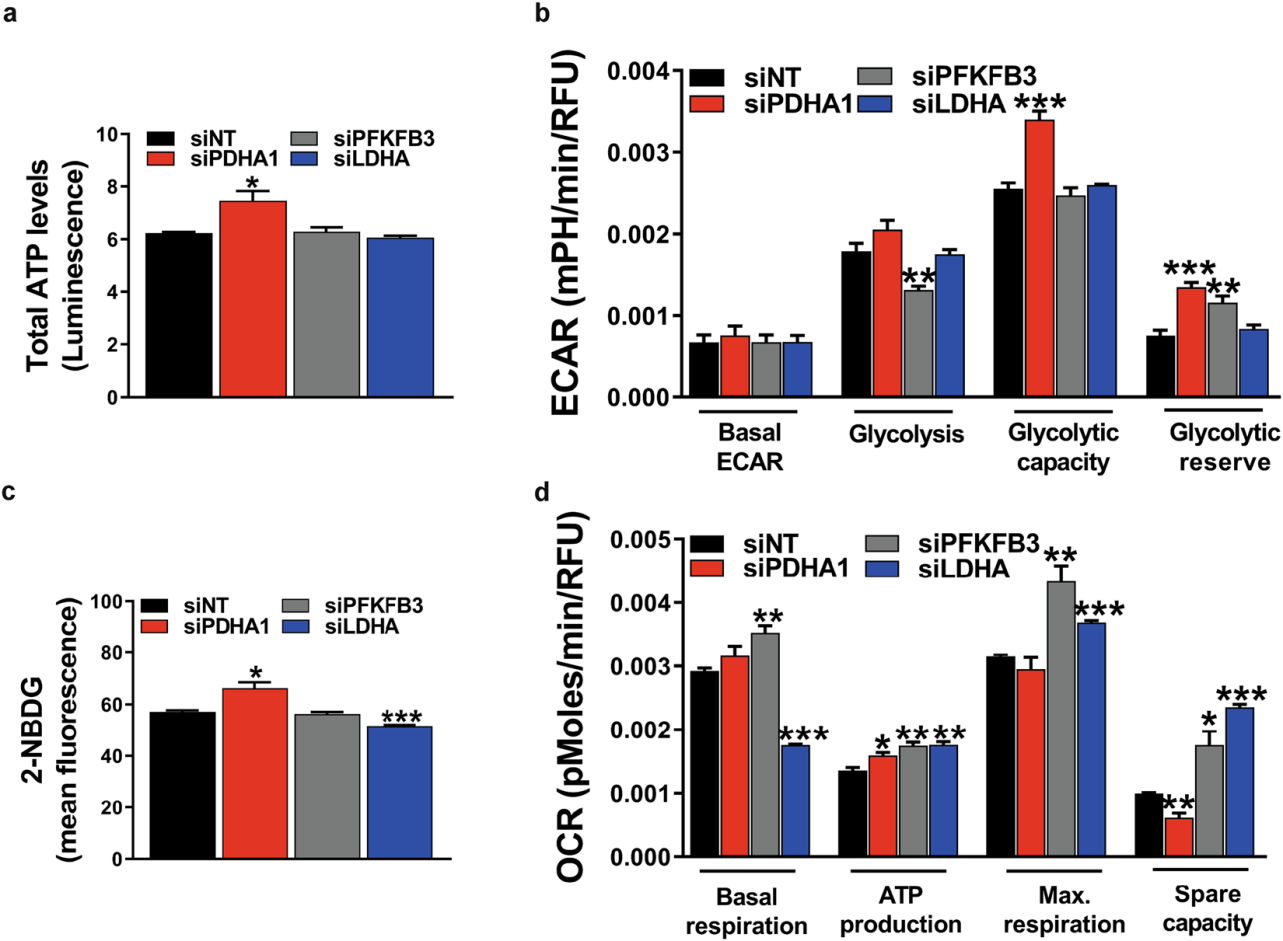
Metabolic flexibility of HUVECs. (**a**) Inhibition of *PDHA1* expression increased total ATP levels (measured as relative luminescence), whereas inhibition of *PFKFB3* or *LDHA* expression did not show effects on total ATP levels at 72 h after siRNA transfection. Assessment of ECAR (**b**) and uptake levels of 2-NBDG (100 µM) (**c**) showed increased levels of glycolysis after inhibition of *PDHA1* expression at 72 h after siRNA transfection. Inhibition of *PFKFB3* expression decreased glucose-induced glycolysis (**b**), but did not have an effect on uptake levels of 2-NBDG (**c**). Inhibition of *LDHA* expression did not affect ECAR (**b**), but lowered 2-NBDG uptake levels (**c**) at 72 h after siRNA transfection. (**d**) siRNA against *PDHA1* induced OCR-linked ATP production, but lowered spare capacity in HUVECs. Silencing of *PFKFB3* gene expression increased OCR. Inhibition of *LDHA* expression lowered basal respiration levels, but increased OCR-linked ATP production, maximal respiration capacity, and spare capacity at 72 h after siRNA transfection. OCR and ECAR measurements were normalized for DNA content (RFU). mpH/min: milli-pH units per minute luminescence was normalized for cell numbers. Results are shown as means ± SEM of experiments with HUVECs of at least 3 donors. *P < 0.05, **P < 0.01, and ***P < 0.001 as compared to siNT (Unpaired Student’s t-test).

Total ATP levels were slightly increased by 1.2-fold after silencing of *PDHA1*, whereas inhibition of *PFKFB3* or *LDHA* expression did not have an effect on total ATP levels (Fig. 4a). The non-metabolizable 2-DG, a glucose analog that inhibits the initial step of glycolysis by its interaction with hexokinase (Fig. 1), was used as a control for total ATP level measurements. It appeared that 2-DG administration reduced total ATP levels by 4-fold after 24 h of treatment (Supplementary Fig. S1h).

Next, metabolic flexibility was studied in more detail by measurements of mitochondrial respiration, glycolysis, and glucose uptake after inhibition of *PDHA1*, *PFKFB3*, or *LDHA* expression, respectively, in HUVECs at 72 h after siRNA transfection. Glycolytic capacity and glycolytic reserve were 1.3-fold and 1.8-fold higher, respectively, in HUVECs transfected with si*PDHA1* as compared to control. No differences were found in basal acidification rates and glucose-induced glycolysis. Silencing of *PFKFB3* resulted in 1.4-fold lower levels of glucose-induced glycolysis as compared to control, whereas *LDHA* silencing did not have an effect on glycolysis (Fig. 4b). Glucose uptake was 16% higher after inhibition of expression of *PDHA1*, whereas *LDHA* silencing resulted in a 11% lower glucose uptake as compared to control. Silencing of *PFKFB3* did not have an effect on glucose uptake (Fig. 4c).

After inhibition of *PDHA1* expression, oxygen consumption rate (OCR)-linked ATP production was increased by 1.2-fold, whereas spare capacity was 1.6-fold lower as compared to control. Inhibition of *PFKFB3* expression resulted in induction of mitochondrial respiration. Basal respiration was 1.7-fold lower after inhibition of *LDHA* expression, whereas OCR-linked ATP production, maximal respiration capacity, and spare capacity were 1.3-fold, 1.2-fold, and 2.4-fold higher, respectively, as compared to control (Fig. 4d).

Taken together, these findings indicate that HUVECs have a flexible metabolism, and that upon blocking the channeling of pyruvate into mitochondrial respiration (by si*PDHA1*), HUVECs lose their spare capacity for mitochondrial respiration but are able to adapt their metabolism by increasing glycolysis. On the other hand, HUVECs increase mitochondrial respiration when glycolysis is inhibited (by si*PFKFB3*).

### Metabolism affects vessel sprouting *in vitro*

**Figure 5:**
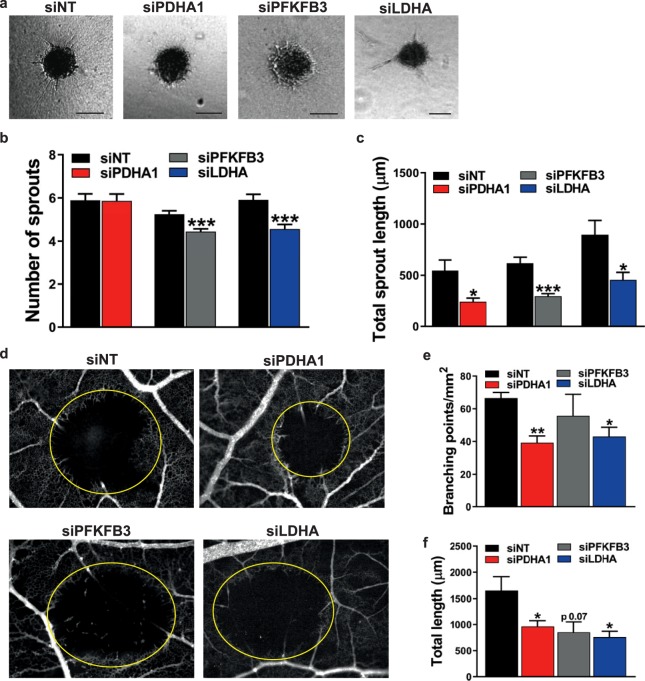
Roles of glycolysis and mitochondrial respiration in angiogenic endothelial sprouting *in vitro* and in the *in vivo* CAM assay. (**a**) Representative images of the *in vitro* spheroid assay after transfection of siRNA against *PDHA1*, *PFKFB3*, *LDHA*, or NT. Scale bar = 100 µm. (**b**) Spheroids showed decreased sprout numbers after inhibition of *PFKFB3* or *LDHA* expression, respectively, at 96 h after siRNA transfection. Inhibition of *PDHA1* expression did not have an effect on the number of sprouts. (**c**) Inhibition of *PDHA1*, *PFKFB3*, or *LDHA* expression, respectively, reduced total sprout length at 96 h after siRNA transfection. (**d**) Representative images of the vascular network in the *in vivo* CAM model after vascular occlusion by photodynamic therapy (yellow-marked zones) and transfection of siRNA against *PDHA1*, *PFKFB3*, *LDHA*, or NT. (**e**) Inhibition of *PDHA1* or *LDHA* expression in the *in vivo* CAM model reduced the number of branching points/mm^2^, respectively, whereas inhibition of *PFKFB3* did not have an effect. (**f**) Total sprout length in the *in vivo* CAM model was reduced after silencing of *PDHA1*, *PFKFB3*, or *LDHA* expression, respectively. Results are shown as means ± SEM of experiments of at least 3 donors. *P < 0.05, **P < 0.01, and ***P < 0.001 as compared to siNT (Unpaired Student’s t-test).

To study the role of metabolism in sprouting angiogenesis *in vitro*, co-culture spheroids^40^ of mixed ECs transfected with siRNA against *PDHA1*, *PFKFB3*, or *LDHA* were embedded in a gel matrix, and HUVEC sprouting was quantified at 96 h after transfection (Fig. 5a). Inhibition of *PDHA1* expression did not have an effect on the number of sprouts (Fig. 5b), whereas *PDHA1* silencing shortened total sprout length as compared to controls by 2.3-fold (Fig. 5c). Inhibition of *PFKFB3* or *LDHA* expression reduced sprout numbers by 1.2-fold and 1.3-fold, respectively, (Fig. 5b) and shortened total sprout length by 2.1-fold and 2-fold, respectively, as compared to controls (Fig. 5c).

Together, these data suggest that both glycolysis and mitochondrial respiration are essential for sprouting angiogenesis *in vitro*.

### Metabolism affects vessel formation in the *in vivo* CAM assay for induced angiogenesis

To further study the role of metabolism during angiogenesis, vascular sprouting in chicken embryos was assessed following vascular occlusion induced by photodynamic therapy using the CAM model. The number of vascular branching points/mm^2^ in the PDT-treated zone and the total length of the sprouts growing in the PDT-treated zone of the CAM were determined after siRNA transfections against *PDHA1*, *PFKFB3*, or *LDHA*, as compared to control (Fig. 5d). Inhibition of *PDHA1* or *LDHA* expression reduced the number of branching points/mm^2^ by 1.7-fold and 1.6-fold, respectively, whereas silencing of *PFKFB3* did not have a significant effect on branching points per mm^2^ (Fig. 5e). Inhibition of the expression of *PDHA1*, *PFKFB3*, or *LDHA* shortened total sprout length by 1.7-fold, 1.9-fold, and 2.2-fold, respectively (Fig. 5f).

These findings indicate that glycolysis as well as mitochondrial respiration are essential for sprouting angiogenesis *in vivo*.

## Discussion

In the present study, we demonstrate that glycolysis is necessary for tip cell differentiation and glucose consumption for mitochondrial ATP production for tip cell survival, whereas glycolysis as well as mitochondrial respiration are essential for the proliferation of non-tip cells. Furthermore, we show that glycolysis as well as mitochondrial respiration are involved in sprouting angiogenesis *in vitro* as well as *in vivo*. Finally, we demonstrate that HUVECs have a flexible metabolism and are able to adapt by using alternative metabolic pathways to generate ATP. In the following sections, we discuss our findings in more detail.

### Glycolysis is necessary for tip cell differentiation

Recent studies indicate that ECs have a relatively high glycolytic activity, which is further increased during angiogenesis^5^. However, we found that ECs are not dependent on a specific metabolic pathway and can adapt their metabolism to microenvironmental circumstances. For example, inhibition of mitochondrial respiration in ECs by specific inhibitors induces a switch to glycolysis, stimulates tip cell differentiation, and inhibits cell proliferation^18,19^. Therefore, unraveling the role of glycolysis and mitochondrial respiration in tip cell/non-tip cell differentiation and in the proliferation of non-tip cells (tip cells do not proliferate) may be key to understand their proposed regulatory role in angiogenesis.

We show here that tip cell differentiation is induced when expression of *PDHA1* is inhibited, and it is reduced when expression of *LDHA* is inhibited. PDHA1 is the gatekeeper enzyme to channel pyruvate from glycolysis into mitochondrial respiration and LDHA is the last enzyme of the glycolytic pathway. Therefore, induction of the tip cell phenotype by inhibition of *PDHA1* may be the result of an intracellular regulatory effect of increased glycolysis. Moreover, we demonstrated that inhibition of *LDHA* expression did not result in apoptotic tip cells. These results show that glycolysis regulates tip cell differentiation and that inhibition of this metabolic pathway blocks the formation of tip cells and that glycolysis is not necessary for survival of existing differentiated tip cells.

### Tip cells need glucose consumption for mitochondrial ATP production for cell survival, and mitochondrial respiration as well as glycolysis are needed for EC proliferation and vascular sprouting

We previously reported that endothelial tip cells show a higher mitochondrial respiration capacity and that tip cells are more dependent on glucose consumption for mitochondrial respiration as compared to non-tip cells^19^. In the present study, we demonstrate that inhibition of *PDHA1* expression induces apoptosis of tip cells, but did not have an effect on non-tip cells. This confirms that tip cells are more dependent on glucose consumption for mitochondrial respiration as compared to non-tip cells.

Vascular development as well as the regression of existing vessels depends on a tightly-regulated balance between proliferation and programmed death of ECs. Endothelial mitochondria are essential for the functional integrity of ECs as they are involved in a wide range of cellular processes including redox signaling^41^. ROS are produced as a byproduct of mitochondrial respiration and are involved in the intracellular signaling associated with cellular processes such as cell proliferation, differentiation, and apoptosis^42^. However, excessive ROS production during cellular stress damages cells and thereby promote cells to go into apoptosis^43^. Several studies indicate that ECs are exquisitely responsive to ROS and that ROS play an important role in vascular diseases, and in the regulation of angiogenesis^42,44^. Besides, nicotinamide adenine dinucleotide phosphate (NADPH)-dependent oxidase 4 (NOX4) is highly expressed in ECs and is essential for basal ROS production and EC proliferation^45,46^. We demonstrate here that silencing of *PDHA1* expression prevents proliferation of HUVECs and inhibits angiogenesis *in vitro* as well as *in vivo*, which may be the result of lower levels of ROS upon inhibition of *PDHA1* expression. Our findings, and particularly those in tip cells, are in agreement with the findings of Garcia-Quintans *et al*.^47,48^ who found that elevated NOX activity and elevated mitochondrial ROS levels induce angiogenesis and suggested that antioxidants directed against mitochondrial ROS levels have therapeutic value in diabetic retinopathy.

Recent studies have shown that lactate is a signaling molecule in angiogenesis. Hypoxia-inducible factor-1α (HIF-1α) levels are stabilized when lactate levels are increased, and angiogenesis is stimulated via VEGF expression^26^. Lactate also increases migration and tube formation of ECs cells by activating the nuclear factor kappa-light-chain-enhancer of activated B cells/interleukin 8 (NFκB/IL8) pathway^49^. The enzyme LDHA has been shown to be essential for angiogenesis in microvascular ECs, and studies in a variety of human cancer types have documented an association between tumor lactate levels and negative clinical outcomes^50,51^. Blocking lactate production reduces the effect of lactate on vascularization, and inhibition of *LDHA* expression reduces vascular network formation in matrigel assays^25,26^. Furthermore, inhibition of *LDHA* expression or inhibition of its activity suppresses cancer cell growth *in vitro* and *in vivo*^23,24^. Consistent with these findings, we demonstrate here that inhibition with siRNA against the glycolytic genes *PFKFB3* or *LDHA*, respectively, prevents the proliferation of HUVECs and inhibits vessel formation *in vitro* as well as *in vivo*. Our EC metabolism studies were confined to siRNA experiments against the glycolytic genes *PFKFB3*, *LDHA* and the mitochondrial gene *PDHA1*. Another approach to study EC metabolism, especially under metabolic stress, is by evaluation of AMP-activated protein kinase (AMPK). AMPK is a metabolic sensor that maintains the balance between ATP production and consumption by sensing cellular energy levels^52–54^. On the other hand, the AMPK system was also found to be involved in cell proliferation^55,56^, making interpretations concerning its role in energy homeostasis difficult.

### ECs have a flexible metabolism and can adapt their metabolism to microenvironmental conditions

Pyruvate can be irreversibly channeled into mitochondria by conversion into acetyl-CoA via the multi-enzyme PDH complex that functions as a gatekeeper between glycolysis and mitochondrial respiration. This occurs in particular under aerobic conditions. Pyruvate can also be reversibly converted into lactate by LDHA (LDHB can reversibly convert lactate into pyruvate). Conversion of pyruvate into lactate occurs in particular under anaerobic conditions^57^. Therefore, cells are unable to convert acetyl-CoA into pyruvate, whereas the conversion of lactate into pyruvate is reversible. To test the metabolic flexibility of ECs, we inhibited the expression of the gene *PDHA1*, which is a critical component for the activity of the multi-enzyme PDH complex^30–33^, and studied glycolysis in more detail. Conversely, we inhibited the expression of the glycolytic genes *PFKFB3* or *LDHA*, consecutively, and studied mitochondrial respiration. We demonstrate here that silencing of *PDHA1* expression increases glucose uptake levels and total cellular ATP levels, but showed no effect on basal acidification rates and glucose-induced glycolysis. In addition to pyruvate, ECs can generate ATP in the presence of oxygen by mitochondrial respiration using alternative substrates such as fatty acids and amino acids (Fig. 1)^19,58^. In line with these findings, we demonstrate that inhibition of *PDHA1* expression has no effect on basal respiration, but has a slightly higher effect on OCR-linked ATP production. This indicates that, upon inhibition of the expression of *PDHA1*, ECs generate their ATP by using substrates other than glucose. ECs are known to exhibit metabolic flexibility that is characterized by shifting from one fuel source to another^19,59,60^. Additionally, metabolic flexibility may allow ECs to shift from mitochondrial respiration to glycolysis to prevent cell damage by avoiding excessive mitochondrial ROS production via mitochondrial respiration^61^.

The glycolytic capacity, glycolytic reserve, mitochondrial respiratory capacity, and mitochondrial spare capacity are measures of the maximum rate of substrate catabolism, which refers to the ability of cells to respond to acutely increased energy demands^62–64^. The plasticity of metabolic capacity over time prevents cells being driven into senescence or cell death^65^. Therefore, the metabolic capacity of ECs provides essential information on energy housekeeping and the effects of pathological conditions. We show that inhibition of *PDHA1* expression increases glycolytic capacity and glycolytic reserve of HUVECs, whereas mitochondrial spare capacity was decreased after *PDHA1* silencing. Overall, this suggests that ECs are not dependent on pyruvate for mitochondrial respiration, and that under basal conditions, ECs do not switch to glycolysis when the channeling of pyruvate into the mitochondrion is blocked, but rather use alternative substrates such as fatty acids and amino acids (Fig. 1). However, when energy demands increase, ECs are able to switch to glycolysis when *PDHA1* expression is inhibited, showing that HUVECs are able to adapt their metabolism to microenvironmental circumstances. These results are consistent with the findings of Wu *et al*.^66^ in cancer cells who showed that inhibition of mitochondrial respiration stimulates glycolysis, and this supports our hypothesis that ECs, like cancer cells, can adapt metabolically, which is called the “glycolytic switch”, when mitochondrial respiration is inhibited.

The conversion of pyruvate into lactate is a reversible reaction, as pyruvate is converted into lactate via LDHA and lactate can be oxidized to pyruvate in a reaction catalyzed by LDHB. Therefore, inhibition of *LDHA* expression can also reduce the amount of pyruvate that is oxidized in the citric acid cycle and mitochondrial respiration. Lactate is known to fuel mitochondrial respiration in cancer cells and ECs^8,67^. We confirm here that inhibition of *LDHA* expression decreases basal respiration levels and glucose uptake levels, but does not affect total ATP levels and glycolytic activity. Inhibition of *LDHA* expression induces OCR-linked ATP production, maximal respiration capacity, and mitochondrial spare capacity. Additionally, inhibition of expression of the glycolytic gene *PFKFB3* partially reduces glycolysis, but increases mitochondrial respiration. Collectively, our outcomes support the findings that the glycolytic end-product lactate is a substrate that can fuel mitochondrial respiration to generate ATP and that ECs, similarly to cancer cells, are able to switch to mitochondrial respiration when glycolysis is inhibited^23,67,68^.

In conclusion, our detailed studies show a complex role of the different metabolic pathways in the specific functions of tip cells and non-tip cells and in the regulation of angiogenesis. Glycolysis regulates initial tip cell formation, and lactate is essential for tip cells to maintain their phenotype. On the other hand, tip cells are more dependent on glucose consumption for their mitochondrial functioning as compared to non-tip cells, whereas glycolysis as well as mitochondrial respiration are essential for the proliferation of non-tip cells. Finally, we demonstrate that the metabolic flexibility of ECs allows the switch between metabolic pathways, e.g. increasing mitochondrial respiration when glycolysis is reduced and vice versa. Together, our findings confirm previous findings on the metabolic flexibility of tip cells and non-tip cells and verify that ECs can adapt to the rapidly changing microenvironment during sprouting angiogenesis^19^, and show that glycolysis as well as mitochondrial respiration are essential for angiogenesis *in vitro* as well as *in vivo*. These important features of ECs may have considerable significance in understanding the complex functioning of metabolism in endothelial tip cells and non-tip cells.

## Material and Methods

### Cell cultures

Primary HUVECs were isolated from umbilical cords, as described earlier^69^, and grown in M199 medium (Gibco, Grand Island, NY, USA) supplemented with 10% heat-inactivated human serum (HS), 10% fetal bovine serum (Gibco), and 1% penicillin-streptomycin-glutamine (Gibco). HUVECs were cultured in T75 culture flasks coated with 2% gelatin (Millipore, Billerica, MA, USA) at 37 °C and 5% CO_2_. Experiments were performed with confluent HUVEC passage 3–4 cells of at least 3 different donors. Human serum and umbilical cords were collected anonymously according to the principles of conduct for research integrity as described in the Medical Treatment Agreement Act in the Civil Code, Book 7 (WGBO BW7:467).

### Identification and isolation of tip cells

The percentage of tip cells in cell cultures was determined as described earlier^35^. Briefly, HUVECs were reverse transfected with siRNA and cells were harvested by TrypLE (Gibco) treatment at 72 h after siRNA transfection. Cells were incubated with CD34-phycoerythrin (1:50; anti-CD34-PE; clone QBend-10; Thermo Scientific, Waltham, MA, USA) in PBS for 30 min at room temperature. Tip cells were identified as CD34^+^ cells using a FACSCalibur (Beckton Dickinson, Franklin Lakes, NJ, USA) and analyzed using FlowJo 6.4.7 software (Tree Star, San Carlos, CA, USA). The fluorescein isothiocyanate (FITC) channel was used to detect autofluorescence. Non-stained and non-treated cells were used as negative controls. For cell sorting experiments, cells were sorted on the basis of CD34 expression (range between 4–20%) with anti-CD34-PE on a Sony SH800Z cell sorter (Sony Biotechnology, Tokyo, Japan). CD34^−^ cells were seeded overnight. After 24 h, CD34^−^ cells were siRNA transfected using the forward transfection method (according to manufacturer’s protocol) and CD34 expression was measured on a FACSCalibur (Beckton Dickinson) at 72 h after siRNA transfection.

### siRNA transfection

Gene silencing in HUVECs was achieved by reversed transfection of siRNA directed against *PFKFB3*, *PDHA1*, *LDHA*, and by non-targeting siRNA (siNT) as a control (20 nM ON-TARGETplus; Dharmacon, Lafayette, CO, USA). According to the manufacturer’s protocol, siRNA was added to OPTI-MEM media (Gibco) with 2.5 µg/ml Dharmafect 1 (Dharmacon) and cells were incubated with starvation medium (M119 medium supplemented with 2% HS). To avoid cytotoxicity, transfection medium was replaced with complete medium at 6 h after siRNA transfection. For each experiment, transfection efficiency was verified using real time quantitative PCR (RT-qPCR) at 72 h after siRNA transfection. Only experiments that showed >70% reduction in mRNA expression were used for further analysis (as shown in Supplementary Fig. S1g). A schematic overview of the relevant genes is shown in Fig. 1.

### RNA isolation, cDNA synthesis, and qPCR

Total RNA was isolated using TRIzol (Invitrogen, Carlsbad, CA, USA) at 24 h after treatment, according to the manufacturer’s instructions. Briefly, total RNA was measured on a NanoDrop (ND-100; NanoDrop Technologies, Wilmington, DE, USA). RNA (1 µg) was DNase I (amplification grade; Invitrogen) treated and reverse transcribed into first strand cDNA using the Maxima first strand cDNA synthesis kit (Thermo Scientific). RT-qPCR was performed on 20x diluted cDNA samples using a CFX96 system (Bio-Rad, Hercules, CA, USA) and specificity of primers was confirmed as described previously^70^. The following tip cell-specific primers were used as previously described^19^: *CD34*, *ANGPT2*, *CXCR4*, *DLL4*, *IGF2*, *NRP2*, *VEGFR2*, and *VEGFR3*. To determine inhibition of gene expression after siRNA transfections the following primers were used: *PDHA1* (Forward 5′-CTCGCAGAGCTTACAGGACGAA-3′; Reverse 5′-GCAGCACCATCGCCATATAAAGTC-3′), *PFKFB3* (Forward 5′-GGAGGCTGTGAAGCAGTACA-3′; Reverse 5′-CAGCTAAGGCACATTGCTTC-3′), *LDHA* (Forward 5′-ACCCAGTTTCCACCATGATT-3′; Reverse 5′-CCCAAAATGCAAGGAACACT-3′). Ct values were converted to arbitrary absolute amounts (2^−Ct^ × 1E^12^) and expressed as fold change as compared to controls. Gene expression data was normalized to tyrosine 3-monooxygenase/tryptophan 5-monooxygenase activation protein and zeta polypeptide (*YHWAZ*), as determined by NormFinder^71^.

### ATP measurements

Total ATP levels (intracellular and extracellular ATP levels) were measured using Cell titer Glo luminescent cell viability assay kit (Promega, Madison, WI, USA) according to the manufacturer’s protocol. The luminescence signal was measured using a microplate reader (CLARIOstar; BMG LABTECH, Ortenberg, Germany) and normalized to the number of cells and the background signal from serum-supplemented medium without cells at 72 h after siRNA transfection. All experiments were performed in triplicate. As a control for total ATP level measurements, cells were treated with 2-deoxyglucose (2-DG) (100 mM; Sigma-Aldrich, St. Louis, MO, USA), a glucose analog that inhibits the initial step of glycolysis by its interaction with hexokinase (Fig. 1), for 24 h in HUVEC medium and luminescence signals were measured as described above.

### *In vitro* glucose uptake

HUVECs were transfected with siRNA and grown in culture plates. To determine glucose uptake levels, cells were incubated with a fluorescent D-glucose analog 2-[N-(7-nitobenz-2-oxa-1,3-diazol-4-yl)-amino]-2-deoxy-D glucose (2-NBDG; Fig. 1) (100 µM; Thermo Scientific) in HUVEC medium for 1 h at 37 °C at 72 h after siRNA transfection. 2-NBDG fluorescence was measured in the FITC channel using a FACSCalibur (Beckton Dickinson) and analyzed using FlowJo 6.4.7 software (Tree Star).

### Measurement of cellular metabolism: flux analysis

OCR, a measure of oxygen utilization of cells, is an important indicator of mitochondrial function^62^. Extracellular acidification rate (ECAR) is a measure of lactic acid levels, formed during the conversion of glucose to lactate during glycolysis^63^. OCR and ECAR were measured using a Seahorse XF96 extracellular flux analyzer (Seahorse Bioscience Europe, Copenhagen, Denmark). HUVECs were reverse transfected with siRNA and were seeded at 40,000 cells per well in Seahorse XF96 polystyrene tissue culture plates (Seahorse Bioscience Europe) and incubated for 72 h. Prior to measurements, cells were incubated in unbuffered DMEM assay medium (Sigma-Aldrich, St. Louis, MO, USA) in a non-CO_2_ incubator at 37 °C for 1 h. Both OCR and ECAR were measured every 4 min with a mixing during 2 min in each cycle, with 4 cycles in total.

DMEM assay medium (Sigma-Aldrich) for OCR measurements contained: glucose (25 mM; Sigma-Aldrich), sodium pyruvate (1 mM; Gibco), and glutamine (2 mM; Gibco). The following inhibitors were injected: oligomycin A (1.5 µM; Cayman Chemical, Ann Arbor, MI, USA), carbonyl cyanide 4-(trifluoromethoxy) phenylhydrazone (FCCP) (1.5 µM; Sigma-Aldrich), antimycin A (2.5 µM; Sigma-Aldrich) and rotenone (1.25 µM; Sigma-Aldrich). This allowed for calculation of OCR-linked ATP production, maximal respiration capacity and spare respiratory capacity. Basal respiration was measured prior to addition of oligomycin A.

The DMEM assay medium (Sigma-Aldrich) for ECAR measurements contained glutamine (2 mM; Gibco) and the following compounds were injected: glucose (10 mM; Sigma-Aldrich), oligomycin A (1.5 µM), and 2-DG (100 mM; Sigma-Aldrich). This allowed for calculation of glycolysis rate, glycolytic capacity, and glycolytic reserve. Basal ECAR was measured prior to addition of glucose.

DNA content was determined using CyQUANT Cell Proliferation Assay (Thermo Scientific) using a microplate reader (CLARIOstar; BMG LABTECH), according to the manufacturer’s instructions. Data is expressed as the mean of measurements in 3 wells and were used to calculate OCR or ECAR normalized to DNA content (relative fluorescence units; RFU).

### Western blotting

HUVECs were transfected with siRNA and harvested after 72 h in lysis buffer (50 mM Tris-HCL, pH 7.4, 150 mM NaCl, 1% Nonidet P-40, 1% Triton X-100, 0.5% sodium deoxycholate, 0.1% SDS) supplemented with protease inhibitor (Roche Diagnostics). For western blot analysis of CD34, 30 µg protein was run on 7% SDS-polyacrylamide gels and transferred onto nitrocellulose membranes (Whatman, Dassel, Germany) by wet blotting. After blocking with 2.5% BSA (Roche Diagnostics) dissolved in Tris-buffered saline (TBS) for 1 h at room temperature, membranes were incubated overnight at 4 °C with primary antibody (Roche Diagnostics) in TBS with 0.05% Tween20 (TBST) and 2.5% BSA. Primary antibodies against CD34 (ab81289; 1:1000; Abcam, Cambridge, UK) and β-actin (1:1000; Sigma-Aldrich) were used as a protein loading control and were detected using secondary antibodies labelled with IRDye700 or IRDye800 (1:1000; LI-COR Biosciences, Lincoln, NE, USA) diluted in TBST containing 2.5% BSA. Blots were scanned on an Odyssey Imager (LI-COR Biosciences) and protein expression was quantified using an Odyssey infrared imaging system and software version 3.0 (LI-COR Biosciences).

### Analysis of apoptosis

Apoptotic cell death in CD34^−^ and CD34^+^ cells were determined at 72 h after siRNA transfection. Cells were stained using FITC-conjugated anti-annexin V (Life technologies, Eugene, OR, USA) and anti-CD34-PE (Thermo Scientific), according to the manufacturer’s instructions. The apoptotic fraction of CD34^−^ and CD34^+^ cells was detected by flow cytometry using a FACSCalibur (Beckton Dickinson) and analyzed using FlowJo 6.4.7 software (Tree Star). As a control for the role of glucose oxidation in tip cell survival, cells were treated with UK5099 (2 µM; Sigma-Aldrich), a compound that blocks the channeling of pyruvate into mitochondrial respiration, for 24 h in HUVEC medium and the percentage of tip cells was measured as described above.

### Cell cycle analysis

Cell proliferation was assessed using a Click-iT^TM^ Plus EdU flow Cytometry Assay Kit (Thermo Scientific), according to the manufacturer’s instructions. Briefly, 10 µM 5-ethynyl-2′-deoxyuridine (EdU) was added to adherent subconfluent HUVEC cultures at 72 h after siRNA transfection and cells were incubated in HUVEC medium for 24 h at 37 °C. Cell cycle analysis was determined by flow cytometry with 488 nm excitation (Beckton Dickinson).

### Spheroid-based *in vitro* angiogenesis model

HUVECs transfected with siRNA were seeded in spheroid medium containing 20% methyl cellulose (Sigma-Aldrich), 78% M199 medium (Gibco), and 2% HS to generate cell spheroids (750 cells/spheroid in 25 µl droplets) at 48 h after transfection. After 18–24 h, spheroids were collected in M119 medium (Gibco) and embedded in a 1 ml gel mixture containing 40% collagen (0.4 mg/ml; Corning, Bedford, MA, USA), 50% methyl cellulose (Sigma-Aldrich), and 2% HS. Spheroids were allowed to sprout for 24 h. Total sprout length and the number of sprouts were quantified of at least 10 spheroids for every condition. All analyses were carried out in a blinded fashion. Quantification was carried out using Neuron-J plug-inn package for Image-J software^72^.

### Chicken chorioallantoic-membrane photodynamic therapy (CAM-PDT) assay

The *in ovo* CAM assay was performed as described earlier^73,74^. Briefly, fertilized chicken eggs were incubated in a hatching incubator with an automatic rotator (Fiem, Italy) and air humidity of 65% at 37 °C. On embryo developmental day (EDD) 3, a small hole was prepared in the eggshell and covered with parafilm (Pechinery, Menasha, WI, USA) to prevent dehydration and possible infections and eggs were returned to the incubator. On EDD 9, vascular occlusion was achieved by Visudyne® photodynamic therapy (PDT). Briefly, 20 µl of Visudyne® (Novartis Pharma Inc., Hettlingen, Switzerland) was intravenously administered to the CAM and allowed to evenly distribute throughout the CAM vasculature for 1 min. Irradiation was achieved using an epi‐fluorescence microscope (Eclipse E 600 FN; Nikon AG, Tokyo, Japan) with x4 and x10 objectives (Plan Apo 4×/0.2, working distance: 20 mm or Plan Fluor 10×/0.3, working distance: 16 mm; Nikon AG), a light dose of 5 J/cm^2^ (λ_ex_ = 420 ± 20 nm) and an irradiance of 35 mW/cm^2^ on a 0.02 cm^2^ area limited by an optical diaphragm within an area defined by a polyethylene ring^75^. siRNA directed against *PFKFB3* (20 µM), *PDHA1* (20 µM), *LDHA* (10 µM), or non-targeted siRNA, premixed with 4-(2-hydroxyethyl)piperazine-1-ethanesulfonic acid (HEPES buffer) and Dharmafect 1 was topically administered directly following PDT. CAMs were visualized and imaged at 24 h after PDT-based vascular occlusion, on EDD10, *in ovo* by means of FITC-dextran (20 kDa, 25 mg/mL; Sigma-Aldrich) epifluorescence angiography, as well as 20 μl of India ink (Pelikan, Witzikon, Switzerland) administered into the extra-embryonic cavity in order to enhance vascular contrast. The total number of sprouts in the PDT-treated zone (number of branching points/mm^2^) and the total sprout length (measured from the edge of the PDT area to the end of the sprout) was quantified using modified ImageJ-based software^76^. The latter was analyzed and quantified manually by two independent observers and carried out in a blinded fashion.

### Statistical analysis

All experiments were performed in HUVECs of at least 3 donors and were performed in duplicate or triplicate. All data were expressed as mean ± standard error of the mean (SEM). GraphPad Prism 6 software was used to assess statistical significance by a two-tailed Student’s t-test. Statistical significance was defined as *p < 0.05, **p < 0.01, ***p < 0.001. To correct for differences between donors, factor correction, as described previously^77^, was used for flow cytometry data, spheroid data, and Seahorse flux data.

## Supplementary information

SUPPLEMENTARY INFO

## Data Availability

The raw datasets generated during and/or analyzed during the current study will be made available upon request to the corresponding author.

## References

[1] Siemerink MJ, Klaassen I, Van Noorden CJ, Schlingemann RO (2013). Endothelial tip cells in ocular angiogenesis: potential target for anti-angiogenesis therapy. J. Histochem. Cytochem..

[2] Geudens I, Gerhardt H (2011). Coordinating cell behaviour during blood vessel formation. Development.

[3] Goveia J, Stapor P, Carmeliet P (2014). Principles of targeting endothelial cell metabolism to treat angiogenesis and endothelial cell dysfunction in disease. EMBO Mol. Med..

[4] Warburg O (1956). On the origin of cancer cells. Science.

[5] De Bock K (2013). Role of PFKFB3-driven glycolysis in vessel sprouting. Cell.

[6] Hanahan D, Weinberg RA (2011). Hallmarks of cancer: the next generation. Cell.

[7] Schoors S (2014). Partial and transient reduction of glycolysis by PFKFB3 blockade reduces pathological angiogenesis. Cell Metab..

[8] Khurshed M, Molenaar RJ, Lenting K, Leenders WP, van Noorden CJF (2017). *In silico* gene expression analysis reveals glycolysis and acetate anaplerosis in IDH1 wild-type glioma and lactate and glutamate anaplerosis in IDH1-mutated glioma. Oncotarget.

[9] Navis AC (2013). Increased mitochondrial activity in a novel IDH1-R132H mutant human oligodendroglioma xenograft model: *in situ* detection of 2-HG and alpha-KG. Acta Neuropathol. Commun.

[10] Talasila KM (2017). The angiogenic switch leads to a metabolic shift in human glioblastoma. Neuro Oncol.

[11] Hui S (2017). Glucose feeds the TCA cycle via circulating lactate. Nature.

[12] Faubert B (2017). Lactate metabolism in human lung tumors. Cell.

[13] Jose C, Bellance N, Rossignol R (2011). Choosing between glycolysis and oxidative phosphorylation: a tumor’s dilemma?. Biochim. Biophys. Acta.

[14] Davidson SM, Duchen MR (2007). Endothelial mitochondria: contributing to vascular function and disease. Circ Res..

[15] Dranka BP, Hill BG, Darley-Usmar VM (2010). Mitochondrial reserve capacity in endothelial cells: the impact of nitric oxide and reactive oxygen species. Free Radic. Biol. Med..

[16] Schleicher M (2008). Prohibitin-1 maintains the angiogenic capacity of endothelial cells by regulating mitochondrial function and senescence. J. Cell Biol..

[17] Al-Mehdi AB (2012). Perinuclear mitochondrial clustering creates an oxidant-rich nuclear domain required for hypoxia-induced transcription. Sci. Signal.

[18] Coutelle O (2014). Embelin inhibits endothelial mitochondrial respiration and impairs neoangiogenesis during tumor growth and wound healing. EMBO Mol. Med..

[19] Yetkin-Arik B (2019). Endothelial tip cells *in vitro* are less glycolytic and have a more flexible response to metabolic stress than non-tip cells. Sci Rep.

[20] van Schaftingen E, Davies DR, Hers HG (1982). Fructose-2,6-bisphosphatase from rat liver. Eur. J. Biochem..

[21] Xu Y (2014). Endothelial PFKFB3 plays a critical role in angiogenesis. Arterioscler. Thromb. Vasc. Biol..

[22] Sonveaux P (2004). Caveolin-1 expression is critical for vascular endothelial growth factor-induced ischemic hindlimb collateralization and nitric oxidemediated angiogenesis. Circ. Res..

[23] Fantin VR, St-Pierre J, Leder P (2006). Attenuation of LDH-A expression uncovers a link between glycolysis, mitochondrial physiology, and tumor maintenance. Cancer Cell.

[24] Le A (2010). Inhibition of lactate dehydrogenase A induces oxidative stress and inhibits tumor progression. Proc. Natl. Acad. Sci. USA.

[25] Parra-Bonilla G, Alvarez DF, Alexeyev M, Vasauskas A, Stevens T (2013). Lactate dehydrogenase a expression is necessary to sustain rapid angiogenesis of pulmonary microvascular endothelium. PloS One.

[26] Sonveaux P (2012). Targeting the lactate transporter MCT1 in endothelial cells inhibits lactate-induced HIF-1 activation and tumor angiogenesis. PloS One.

[27] Beckert S (2006). Lactate stimulates endothelial cell migration. Wound Repair Regen.

[28] Kumar VB, Viji RI, Kiran MS, Sudhakaran PR (2007). Endothelial cell response to lactate: implication of PAR modification of VEGF. Cell Physiol.

[29] Bresters TW, de Abreu RA, de Kok A, Visser J, Veeger C (1975). The pyruvate-dehydrogenase complex from Azotobacter vinelandii. Eur. J. Biochem..

[30] Brivet M (2005). First characterization of a large deletion of the PDHA 1 gene. Mol. Genet. Metab..

[31] Cameron JM, Levandovskiy V, Mackay N, Tein I, Robinson BH (2004). Deficiency of pyruvate dehydrogenase caused by novel and known mutations in the E1alpha subunit. Am. J. Med. Genet. A.

[32] Patel KP, O’Brien TW, Subramony SH, Shuster J, Stacpoole PW (2012). The spectrum of pyruvate dehydrogenase complex deficiency: clinical, biochemical and genetic features in 371 patients. Mol. Genet. Metab..

[33] Steller J, Gargus JJ, Gibbs LH, Hasso AN, Kimonis VE (2014). Mild phenotype in a male with pyruvate dehydrogenase complex deficiency associated with novel hemizygous in-frame duplication of the E1alpha subunit gene (PDHA1). Neuropediatrics.

[34] Kim JW, Tchernyshyov I, Semenza GL, Dang CV (2006). HIF-1-mediated expression of pyruvate dehydrogenase kinase: a metabolic switch required for cellular adaptation to hypoxia. Cell Metab..

[35] Siemerink MJ (2012). CD34 marks angiogenic tip cells in human vascular endothelial cell cultures. Angiogenesis.

[36] Dallinga MG (2018). IGF2 and IGF1R identified as novel tip cell genes in primary microvascular endothelial cell monolayers. Angiogenesis.

[37] Jakobsson L (2010). Endothelial cells dynamically compete for the tip cell position during angiogenic sprouting. Nat. Cell Biol..

[38] Elmasri H (2009). Fatty acid binding protein 4 is a target of VEGF and a regulator of cell proliferation in endothelial cells. FASEB J..

[39] Leopold JA (2003). Glucose-6-phosphate dehydrogenase modulates vascular endothelial growth factor-mediated angiogenesis. J. Biol. Chem..

[40] Korff T, Augustin HG (1998). Integration of endothelial cells in multicellular spheroids prevents apoptosis and induces differentiation. J. Cell Biol..

[41] Widlansky ME, Gutterman DD (2011). Regulation of endothelial function by mitochondrial reactive oxygen species. Antioxid. Redox Signal.

[42] Xia C (2007). Reactive oxygen species regulate angiogenesis and tumor growth through vascular endothelial growth factor. Cancer Res..

[43] Kluge MA, Fetterman JL, Vita JA (2013). Mitochondria and endothelial function. Circ. Res..

[44] Lee Y, Son JY, Kang JI, Park KM, Park KD (2018). Hydrogen peroxide-releasing hydrogels for enhanced endothelial cell activities and neovascularization. ACS Appl. Mater Interfaces.

[45] Petry A (2006). NOX2 and NOX4 mediate proliferative response in endothelial cells. Antioxid. Redox Signal.

[46] Abid MR, Kachra Z, Spokes KC, Aird WC (2000). NADPH oxidase activity is required for endothelial cell proliferation and migration. FEBS Lett.

[47] Garcia-Quintans N (2016). Oxidative stress induces loss of pericyte coverage and vascular instability in PGC-1alpha-deficient mice. Angiogenesis.

[48] Garcia-Quintans N (2016). Regulation of endothelial dynamics by PGC-1alpha relies on ROS control of VEGF-A signaling. Free Radic. Biol. Med..

[49] Vegran F, Boidot R, Michiels C, Sonveaux P, Feron O (2011). Lactate influx through the endothelial cell monocarboxylate transporter MCT1 supports an NF-kappaB/IL-8 pathway that drives tumor angiogenesis. Cancer Res..

[50] Brizel DM (2001). Elevated tumor lactate concentrations predict for an increased risk of metastases in head-and-neck cancer. Int. J. Radiat. Oncol. Biol., Phys..

[51] Walenta S, Schroeder T, Mueller-Klieser W (2004). Lactate in solid malignant tumors: potential basis of a metabolic classification in clinical oncology. Curr. Med .Chem.

[52] Hardie DG (2007). AMP-activated/SNF1 protein kinases: conserved guardians of cellular energy. Nat. Rev. Mol. Cell Biol..

[53] Kahn BB, Alquier T, Carling D, Hardie DG (2005). AMP-activated protein kinase: Ancient energy gauge provides clues to modern understanding of metabolism. Cell Metab..

[54] Steinberg GR, Kemp BE (2009). AMPK in Health and Disease. Physiol. Rev..

[55] Jones RG (2005). AMP-activated protein kinase induces a p53-dependent metabolic checkpoint. Mol. Cell.

[56] Rattan R, Giri S, Singh AK, Singh I (2005). 5-Aminoimidazole-4-carboxamide-1-beta-D-ribofuranoside inhibits cancer cell proliferation *in vitro* and *in vivo* via AMP-activated protein kinase. J. Biol. Chem..

[57] Tennant DA, Duran RV, Boulahbel H, Gottlieb E (2009). Metabolic transformation in cancer. Carcinogenesis.

[58] Krutzfeldt A, Spahr R, Mertens S, Siegmund B, Piper HM (1990). Metabolism of exogenous substrates by coronary endothelial cells in culture. J. Mol. Cell Cardiol..

[59] Dagher Z, Ruderman N, Tornheim K, Ido Y (2001). Acute regulation of fatty acid oxidation and amp-activated protein kinase in human umbilical vein endothelial cells. Circ. Res..

[60] Schoors S (2015). Fatty acid carbon is essential for dNTP synthesis in endothelial cells. Nature.

[61] Liemburg-Apers DC, Willems PH, Koopman WJ, Grefte S (2015). Interactions between mitochondrial reactive oxygen species and cellular glucose metabolism. Arch. Toxicol.

[62] Divakaruni AS, Paradyse A, Ferrick DA, Murphy AN, Jastroch M (2014). Analysis and interpretation of microplate-based oxygen consumption and pH data. Meth .Enzymol.

[63] Ferrick DA, Neilson A, Beeson C (2008). Advances in measuring cellular bioenergetics using extracellular flux. Drug Discov. Today.

[64] Mookerjee SA, Nicholls DG, Brand MD (2016). Determining maximum glycolytic capacity using extracellular flux measurements. PloS One.

[65] Desler C (2012). Is there a link between mitochondrial reserve respiratory capacity and aging?. J. Aging Res.

[66] Wu M (2007). Multiparameter metabolic analysis reveals a close link between attenuated mitochondrial bioenergetic function and enhanced glycolysis dependency in human tumor cells. Am. J. Physiol. Cell Physiol..

[67] Polet F, Feron O (2013). Endothelial cell metabolism and tumour angiogenesis: glucose and glutamine as essential fuels and lactate as the driving force. J. Intern. Med..

[68] Sonveaux P (2008). Targeting lactate-fueled respiration selectively kills hypoxic tumor cells in mice. J. Clin .Invest..

[69] Crampton, S. P., Davis, J. & Hughes, C. C. Isolation of human umbilical vein endothelial cells (HUVEC). *J. Vis. Exp*. **183**, 10.3791/183 (2007). 10.3791/183 PMC2576276 18978951

[70] Klaassen I (2009). Altered expression of genes related to blood-retina barrier disruption in streptozotocin-induced diabetes. Exp. Eye Res..

[71] Andersen CL, Jensen JL, Orntoft TF (2004). Normalization of real-time quantitative reverse transcription-PCR data: a model-based variance estimation approach to identify genes suited for normalization, applied to bladder and colon cancer data sets. Cancer Res..

[72] Chieco P, Jonker A, De Boer BA, Ruijter JM, Van Noorden CJ (2013). Image cytometry: protocols for 2D and 3D quantification in microscopic images. Progr Histochem Cytochem.

[73] Nowak-Sliwinska P (2018). Consensus guidelines for the use and interpretation of angiogenesis assays. Angiogenesis.

[74] Nowak-Sliwinska P, Segura T, Iruela-Arispe ML (2014). The chicken chorioallantoic membrane model in biology, medicine and bioengineering. Angiogenesis.

[75] Lim SH (2010). The neovessel occlusion efficacy of 15-hydroxypurpurin-7-lactone dimethyl ester induced with photodynamic therapy. Photochem. Photobiol.

[76] Weiss A (2015). Rapid optimization of drug combinations for the optimal angiostatic treatment of cancer. Angiogenesis.

[77] Ruijter JM (2006). Factor correction as a tool to eliminate between-session variation in replicate experiments: application to molecular biology and retrovirology. Retrovirology.

