# Supplementary material for: The role of glycolysis and mitochondrial respiration in the formation and functioning of endothelial tip cells during angiogenesis
Source: Sci Rep. 2019 Aug 30;9:12608. doi: 10.1038/s41598-019-48676-2 (PMC6717205; doi:10.1038/s41598-019-48676-2)
Supplement: Supplementary file 1 — SUPPLEMENTARY INFO [file 41598_2019_48676_MOESM1_ESM.pdf]

The role of glycolysis and mitochondrial respiration in the formation and functioning of endothelial tip cells during angiogenesis

Bahar Yetkin-Arik [1,2], Ilse M.C. Vogels [1,2], Patrycja Nowak-Sliwinska [3], Andrea Weiss [3], Riekelt H. Houtkooper [4], Cornelis J.F. Van Noorden [2,5], Ingeborg Klaassen [1,2#*], Reinier O. Schlingemann [1,6#]

[1] Ocular Angiogenesis Group, Department of Ophthalmology and [2] Department of Medical Biology, Amsterdam Cardiovascular Sciences, Cancer Center Amsterdam, Amsterdam UMC, University of Amsterdam, Meibergdreef 9, Amsterdam, The Netherlands.

[3] Molecular Pharmacology Group, School of Pharmaceutical Sciences, Faculty of Sciences, University of Geneva, University of Lausanne, Geneva, Switzerland.

[4] Laboratory Genetic Metabolic Diseases, Amsterdam Gastroenterology and Metabolism, Amsterdam Cardiovascular Sciences, Amsterdam UMC, University of Amsterdam, Meibergdreef 9, Amsterdam, The Netherlands.

[5] Department of Genetic Toxicology and Cancer Biology, National Institute of Biology, Ljubljana, Slovenia.

[6] Department of Ophthalmology, University of Lausanne, Jules-Gonin Eye Hospital, Fondation Asile des Aveugles, Lausanne, Switzerland.

[#] Authors contributed equally.

* Correspondence:

Dr. Ingeborg Klaassen, Department of Medical Biology, Amsterdam UMC, Academic Medical Center, Meibergdreef 15, Room L3-154, 1105 AZ Amsterdam, The Netherlands, E-mail: i.klaassen@amc.uva.nl.

Supplementary figures

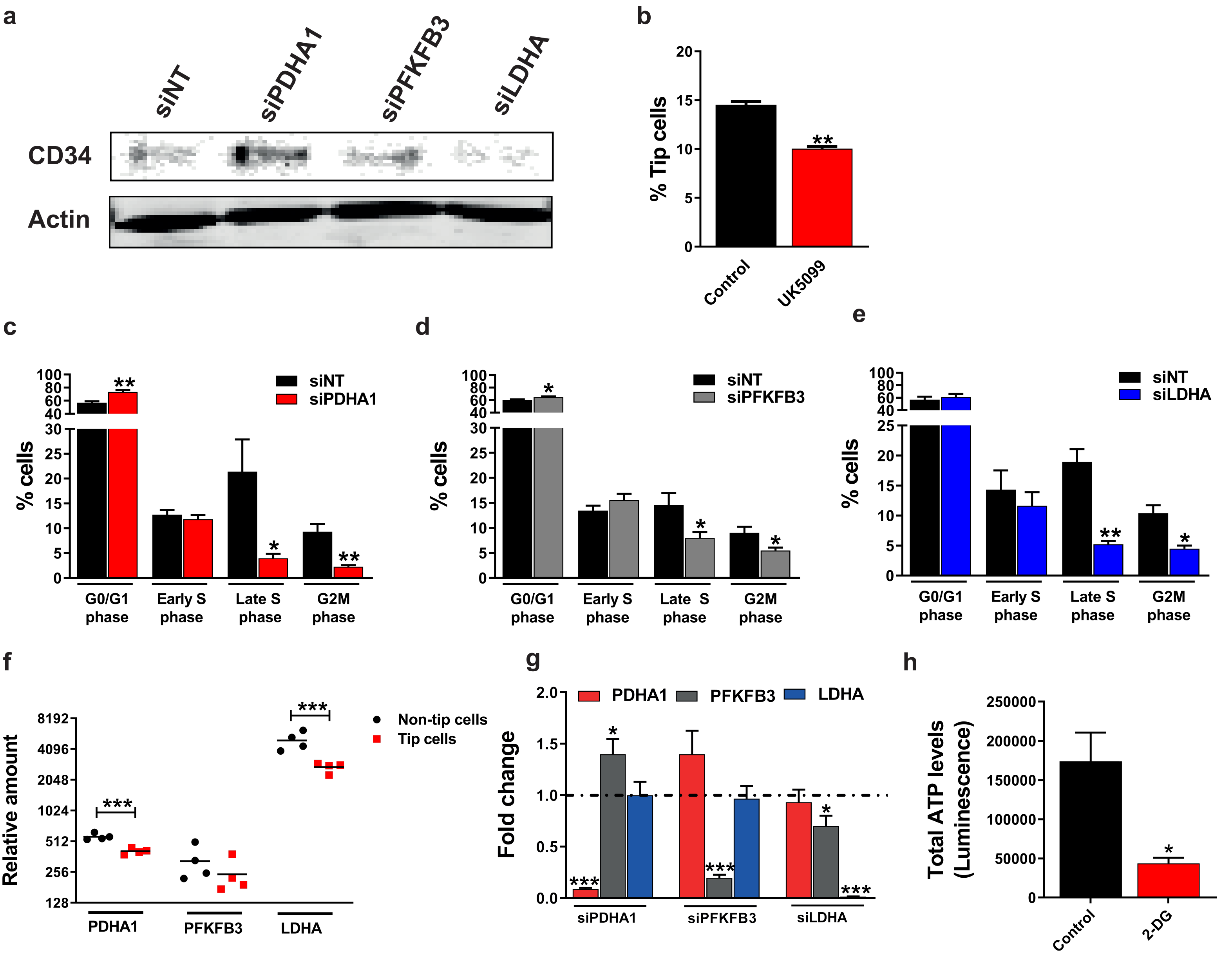

Fig. S1

a Cropped western blot images. Western blot analysis of CD34 protein with actin as internal control after transfection of siRNA against *PDHA1*, *PFKFB3*, or *LDHA*, respectively, confirmed the findings of Fig. 2a-e. Full length blots with multiple exposures for actin and CD34 protein are presented in Supplementary Fig. S2a and Fig. S2b, respectively. b Treatment of HUVECs with UK5099 reduced the number of tip cells at 24 h after treatment. Cell proliferation assay of HUVECs expressed as percentages of non-proliferating cells in the G0/G1 phase, and proliferating cells in early S phase, late S phase and G2M phase after flow cytometric analysis of fluorescence of incorporated EdU at 72 h after transfection of siRNA against *PDHA1*, *PFKFB3*, or *LDHA*, respectively. **c-e** Inhibition of the expression of *PDHA1*, *PFKFB3*, or *LDHA* lowered percentages of HUVECs in the late S phase and G2M phase and inhibition of the expression of *PDHA1* or *PFKFB3* increased the percentages of HUVECs in the G0/G1 phase, respectively (**c-d**). **f** Expression levels of the genes *PDHA1* and *LDHA*, but not of *PFKFB3*, in sorted fractions of tip cells and non-tip cells were lower in tip cells as compared to non-tip cells. **g** Inhibition of *PDHA1* expression increased expression levels of *PFKFB3*. Inhbition of *PFKFB3* expression did not have an effect on *PDHA1* and *LDHA* mRNA expression levels. Silencing of *LDHA* expression downregulated *PFKFB3* expression levels and showed no effect on *PDHA1* expression. **h** Measurements of total ATP levels (determined as relative luminescence) after 2-DG treatment revealed reduced total ATP levels at 24 h of treatment. Results are shown as means ± SEM of experiments with HUVECs of at least 3 donors.
* P<0.05, ** P<0.01, and *** P<0.001 as compared to siNT (Unpaired Student's t-test).

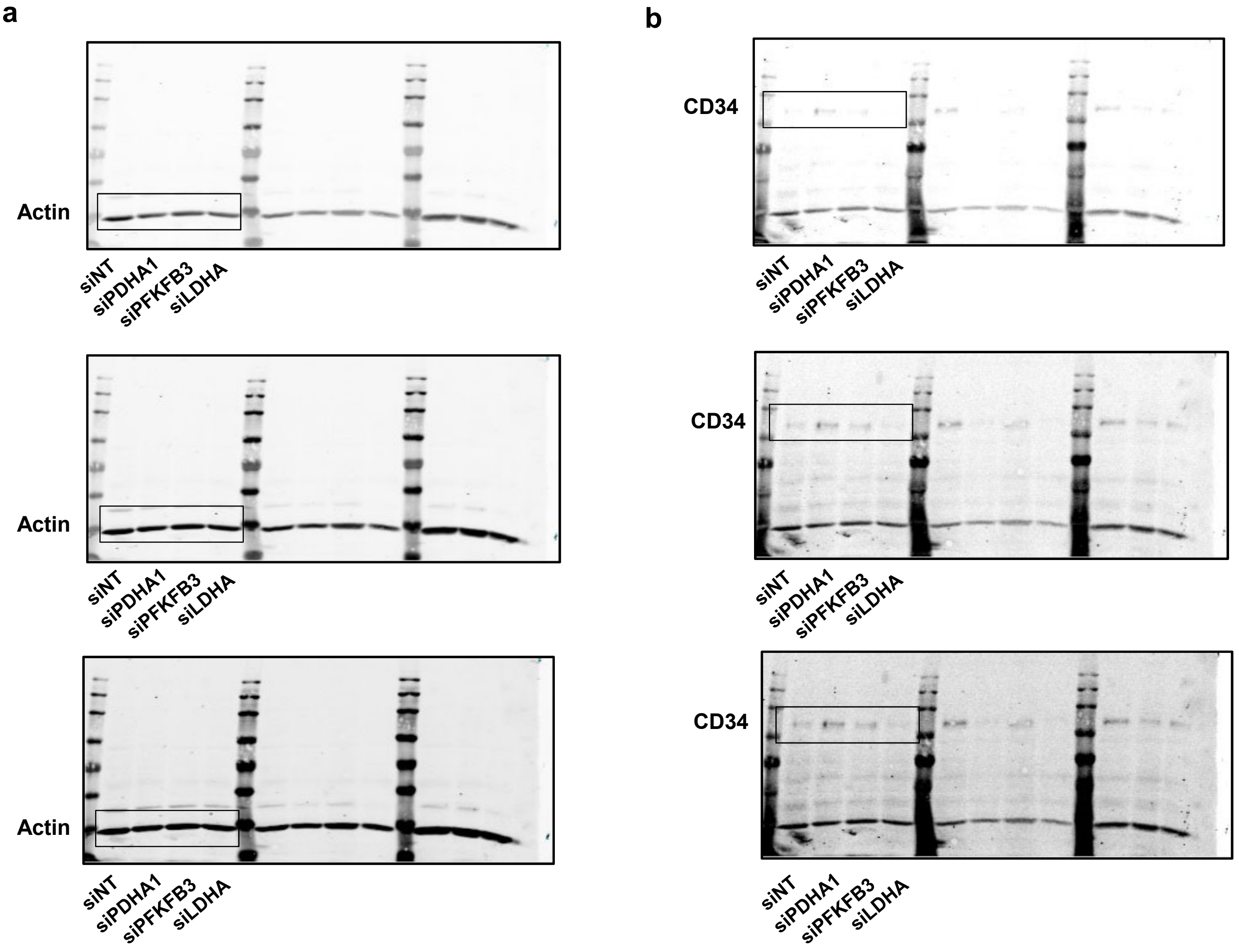

Fig. S2

Full length blots of cropped western blot images (Supplementary Fig. S1g) with multiple exposures for actin (**a**) and CD34 protein (**b**) after transfection of siRNA against *PDHA1*, *PFKFB3*, or *LDHA*.
